# [4,4′-(Ethane-1,2-diyldinitrilo)­bis­(pent-2-en-2-olato)]copper(II) 0.25-hydrate

**DOI:** 10.1107/S1600536812017102

**Published:** 2012-04-25

**Authors:** Muhammad Aslam, Itrat Anis, Nighat Afza, Ajaz Hussain, Waseem Ahmed, Muhammad Nadeem Arshad

**Affiliations:** a Pakistan Council of Scientific and Industrial Research Laboratories Complex, Karachi 75280, Pakistan; b Department of Chemistry, University of Karachi, Karachi 75270, Pakistan; c Department of Chemistry, GC University, Faisalabad, Pakistan; d Department of Biochemistry, Federal Urdu University of Arts Science and Technology, Gulshan-e-Iqbal Campus, Karachi, Pakistan; e Department of Chemistry, University of Gujrat (Hafiz Hayat campus), Gujrat 50781, Pakistan

## Abstract

In the title compound, [Cu(C_12_H_18_N_2_O_2_)]·0.25H_2_O, the coordination of the *O*,*N*,*N*′,*O*′-tetra­dentate ligand results in a *cis*-CuN_2_O_2_ square-planar geometry for the metal ion and the presence of two six-membered and one five-membered chelate rings. The complete complex mol­ecule is close to planar (r.m.s. deviation = 0.047 Å). The uncoordinated water mol­ecule (O-atom site symmetry 2) was modelled as half occupied. In the crystal, C—H⋯O_w_ and O_w_—H⋯O (w = water) hydrogen bonds link the components into layers parallel to *ab* plane.

## Related literature

For background to Schiff bases and their complexes, see: Aslam *et al.* (2012 ▶).
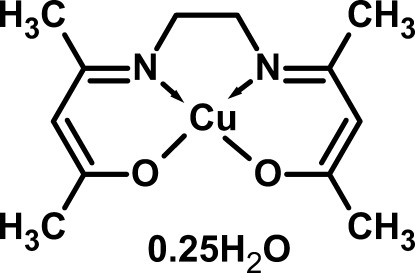


## Experimental

### 

#### Crystal data

[Cu(C_12_H_18_N_2_O_2_)]·0.25H_2_O
*M* *_r_* = 290.33
Orthorhombic, 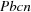

*a* = 17.0029 (7) Å
*b* = 8.0198 (3) Å
*c* = 19.6532 (7) Å
*V* = 2679.91 (18) Å^3^
*Z* = 8
Mo *K*α radiation
μ = 1.62 mm^−1^
*T* = 296 K
0.24 × 0.21 × 0.13 mm

#### Data collection

Bruker Kappa APEXII CCD diffractometer
Absorption correction: multi-scan (*SADABS*; Bruker, 2007 ▶) *T*_min_ = 0.697, *T*_max_ = 0.817
22704 measured reflections
3338 independent reflections
1745 reflections with *I* > 2σ(*I*)
*R*_int_ = 0.042

#### Refinement

*R*[*F* ^2^ > 2σ(*F* ^2^)] = 0.060
*wR*(*F* ^2^) = 0.190
*S* = 1.07
3338 reflections
166 parameters
1 restraint
H atoms treated by a mixture of independent and constrained refinement
Δρ_max_ = 0.56 e Å^−3^
Δρ_min_ = −0.39 e Å^−3^

### 

Data collection: *APEX2* (Bruker, 2007 ▶); cell refinement: *SAINT* (Bruker, 2007 ▶); data reduction: *SAINT*; program(s) used to solve structure: *SHELXS97* (Sheldrick, 2008 ▶); program(s) used to refine structure: *SHELXL97* (Sheldrick, 2008 ▶); molecular graphics: *PLATON* (Spek, 2009 ▶); software used to prepare material for publication: *WinGX* (Farrugia, 1999 ▶) and *PLATON*.

## Supplementary Material

Crystal structure: contains datablock(s) I, global. DOI: 10.1107/S1600536812017102/hb6739sup1.cif

Structure factors: contains datablock(s) I. DOI: 10.1107/S1600536812017102/hb6739Isup2.hkl

Additional supplementary materials:  crystallographic information; 3D view; checkCIF report

## Figures and Tables

**Table 1:** Selected bond lengths (Å)

Cu1—O2	1.897 (4)
Cu1—O1	1.901 (4)
Cu1—N2	1.922 (4)
Cu1—N1	1.926 (4)

**Table 2:** Hydrogen-bond geometry (Å, °)

*D*—H⋯*A*	*D*—H	H⋯*A*	*D*⋯*A*	*D*—H⋯*A*
C12—H12*A*⋯O1*W*^i^	0.96	2.58	3.466 (9)	154
C6—H6*B*⋯O1*W*^i^	0.97	2.44	3.303 (11)	149
O1*W*—H1*WA*⋯O2	0.79 (2)	2.29 (14)	2.862 (8)	130 (13)

Symmetry code: (i) 
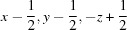
.

**Fig. 1.**
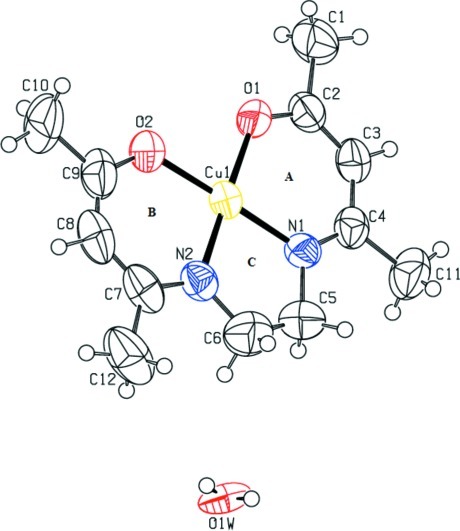
The molecular structure of (I) with 50% displacement ellipsoids.

**Fig. 2.**
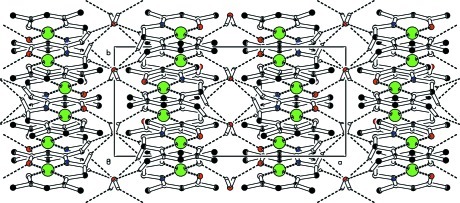
A view of the unit cell packing showing two dimensional hydrogen bonding network through dashed lines.

## supplementary crystallographic information

### Comment

Herein we report the crystal structure of title compound, (I), which is a Cu complex of schiff base in countinuation of our studies on synthesis and metal complexation of schiff bases (Aslam *et al.*, 2012). The copper metal bonded to the ligand 4,4'-(ethane-1,2-diyldinitrilo)bispent-2-en-2-ol through Cu—O covelant & Cu—N coordinate covelant bonds in such a way that it causes to produce two six membered rings (C2/C3/C4/Cu1/N1/O1) "A" & (C7/C8/C9/Cu1/N2/O2) "B" and a five membered ring (C5/C6/N1/Cu1/N2) "C". All these three rings are almost planer with the r. m. s. deviation of 0.0114 Å, 0.0061 Å and 0.0273 Å respectively. The molecule as a whole is slightly twisted as the five membered ring is oriented at dihedral angle of 2.54 (23)° and 3.34 (23)° with respect to six membered rings "A" and "B". Both of six membered rings are twisted at angle of 3.47 (16)° which showes the slight nonplaner behaviour of the molecule.

Moreover, a half water molecule have also been identified during refinement which links the molecules in two dimensional network through O—H···O and C—H···O interactions along *a* & *b* axes (Table. 2, Fig. 2).

### Experimental

A methanolic solution of copper (II) chloride dihydrated (1 mol) (5 ml) was slowly added to a methanolic solution of 4-{[2-((1-methyl-3-oxobutylidene)amino)ethyl]imino}-2-pentanone (1 mol) (100 ml) and refluxed with stirring for 45 min. The pH was gradually raised to achieve the suitable pH for the formulation of the complex by the drop wise addition of 1 *M* NaOH solution. Now the reaction mixture was refluxed with stirring for 90 min. After cooling, the mixture was concentrated to one third under reduced pressure. The concentrated reaction mixture was kept at room temperature and black crystals were obtained after six days. The crystalline product was collected, washed with cooled methanol, recrystallized from ethylacetate and methanol (1:1) mixture and dried to afford the title compound in 73.1% yield. Slow evaporation of a methanol solution afforded dark brown blocks of (I).

### Refinement

All the C—H and H-atoms were positioned with idealized geometry with C—H = 0.93 Å for aromatic, C—H = 0.97 Å for methylene & C—H = 0.96 Å for methyl group and were refined using a riding model with *U*_iso_(H) = 1.2 *U*_eq_(C) for aromatic & methylene and *U*_iso_(H) = 1.5 *U*_eq_(C) for methyl carbon atoms. The O—H = 0.79 (2) Å & O–H 0.95 (2) Å H atoms were refined using fourier map with *U*_iso_(H) = 1.5 *U*_eq_(O).

### Crystal data

**Table:**

[Cu(C_12_H_18_N_2_O_2_)]·0.25H_2_O	*F*(000) = 1212
*M**_r_* = 290.33	*D*_x_ = 1.439 Mg m^−^^3^
Orthorhombic, *P**b**c**n*	Mo *K*α radiation, λ = 0.71073 Å
Hall symbol: -P 2n 2ab	Cell parameters from 5532 reflections
*a* = 17.0029 (7) Å	θ = 2.4–25.9°
*b* = 8.0198 (3) Å	µ = 1.62 mm^−^^1^
*c* = 19.6532 (7) Å	*T* = 296 K
*V* = 2679.91 (18) Å^3^	Block, dark brown
*Z* = 8	0.24 × 0.21 × 0.13 mm

### Data collection

**Table:**

Bruker Kappa APEXII CCD diffractometer	3338 independent reflections
Radiation source: fine-focus sealed tube	1745 reflections with *I* > 2σ(*I*)
Graphite monochromator	*R*_int_ = 0.042
φ and ω scans	θ_max_ = 28.3°, θ_min_ = 2.8°
Absorption correction: multi-scan (*SADABS*; Bruker, 2007)	*h* = −22→22
*T*_min_ = 0.697, *T*_max_ = 0.817	*k* = −8→10
22704 measured reflections	*l* = −22→26

### Refinement

**Table:**

Refinement on *F*^2^	Primary atom site location: structure-invariant direct methods
Least-squares matrix: full	Secondary atom site location: difference Fourier map
*R*[*F*^2^ > 2σ(*F*^2^)] = 0.060	Hydrogen site location: inferred from neighbouring sites
*wR*(*F*^2^) = 0.190	H atoms treated by a mixture of independent and constrained refinement
*S* = 1.07	*w* = 1/[σ^2^(*F*_o_^2^) + (0.0608*P*)^2^ + 6.2772*P*] where *P* = (*F*_o_^2^ + 2*F*_c_^2^)/3
3338 reflections	(Δ/σ)_max_ < 0.001
166 parameters	Δρ_max_ = 0.56 e Å^−^^3^
1 restraint	Δρ_min_ = −0.39 e Å^−^^3^

### Special details

**Table:**

Geometry. All s.u.'s (except the s.u. in the dihedral angle between two l.s. planes) are estimated using the full covariance matrix. The cell s.u.'s are taken into account individually in the estimation of s.u.'s in distances, angles and torsion angles; correlations between s.u.'s in cell parameters are only used when they are defined by crystal symmetry. An approximate (isotropic) treatment of cell s.u.'s is used for estimating s.u.'s involving l.s. planes.
Refinement. Refinement of *F*^2^ against ALL reflections. The weighted *R*-factor *wR* and goodness of fit *S* are based on *F*^2^, conventional *R*-factors *R* are based on *F*, with *F* set to zero for negative *F*^2^. The threshold expression of *F*^2^ > 2σ(*F*^2^) is used only for calculating *R*-factors(gt) *etc*. and is not relevant to the choice of reflections for refinement. *R*-factors based on *F*^2^ are statistically about twice as large as those based on *F*, and *R*- factors based on ALL data will be even larger.

### Fractional atomic coordinates and isotropic or equivalent isotropic displacement parameters (Å^2^)

**Table:**

	*x*	*y*	*z*	*U*_iso_*/*U*_eq_	Occ. (<1)
Cu1	0.28720 (4)	0.12613 (9)	0.19272 (3)	0.0578 (3)	
O1	0.3694 (2)	0.1823 (5)	0.13133 (18)	0.0729 (11)	
O2	0.3675 (2)	0.0653 (6)	0.25492 (19)	0.0791 (12)	
N1	0.2057 (2)	0.2001 (5)	0.1317 (2)	0.0557 (10)	
N2	0.2037 (3)	0.0604 (5)	0.2529 (2)	0.0609 (11)	
C1	0.4377 (4)	0.2870 (10)	0.0361 (3)	0.096 (2)	
H1A	0.4698	0.1885	0.0365	0.144*	
H1B	0.4650	0.3758	0.0589	0.144*	
H1C	0.4272	0.3189	−0.0101	0.144*	
C2	0.3606 (4)	0.2520 (7)	0.0726 (3)	0.0662 (15)	
C3	0.2912 (4)	0.2910 (7)	0.0431 (3)	0.0670 (15)	
H3	0.2932	0.3401	0.0002	0.080*	
C4	0.2160 (3)	0.2638 (6)	0.0715 (3)	0.0596 (13)	
C5	0.1275 (4)	0.1746 (10)	0.1597 (4)	0.094 (2)	
H5A	0.1025	0.2822	0.1659	0.113*	
H5B	0.0963	0.1117	0.1273	0.113*	
C6	0.1280 (4)	0.0880 (11)	0.2234 (4)	0.109 (3)	
H6A	0.0964	0.1507	0.2555	0.131*	
H6B	0.1027	−0.0193	0.2169	0.131*	
C7	0.2129 (4)	−0.0013 (8)	0.3139 (3)	0.0741 (17)	
C8	0.2866 (5)	−0.0314 (8)	0.3429 (3)	0.084 (2)	
H8	0.2871	−0.0793	0.3859	0.100*	
C9	0.3573 (5)	0.0023 (9)	0.3145 (3)	0.0825 (18)	
C10	0.4323 (5)	−0.0375 (12)	0.3520 (4)	0.126 (3)	
H10A	0.4631	0.0622	0.3569	0.189*	
H10B	0.4617	−0.1189	0.3269	0.189*	
H10C	0.4199	−0.0811	0.3962	0.189*	
C11	0.1458 (4)	0.3136 (9)	0.0287 (3)	0.094 (2)	
H11A	0.1143	0.3925	0.0534	0.141*	
H11B	0.1149	0.2166	0.0186	0.141*	
H11C	0.1637	0.3633	−0.0129	0.141*	
C12	0.1418 (5)	−0.0453 (10)	0.3561 (4)	0.116 (3)	
H12A	0.1105	−0.1259	0.3323	0.174*	
H12B	0.1111	0.0533	0.3642	0.174*	
H12C	0.1585	−0.0912	0.3988	0.174*	
O1W	0.5000	0.2851 (13)	0.2500	0.070 (3)	0.50
H1WA	0.484 (9)	0.205 (9)	0.269 (6)	0.105*	0.50

### Atomic displacement parameters (Å^2^)

**Table:**

	*U*^11^	*U*^22^	*U*^33^	*U*^12^	*U*^13^	*U*^23^
Cu1	0.0602 (4)	0.0703 (5)	0.0430 (4)	0.0012 (4)	−0.0011 (3)	−0.0032 (3)
O1	0.054 (2)	0.116 (3)	0.049 (2)	−0.002 (2)	−0.0047 (17)	0.005 (2)
O2	0.071 (2)	0.117 (3)	0.049 (2)	0.011 (2)	−0.0059 (19)	0.006 (2)
N1	0.053 (2)	0.059 (2)	0.056 (2)	0.002 (2)	0.003 (2)	−0.010 (2)
N2	0.068 (3)	0.060 (3)	0.055 (3)	−0.006 (2)	0.004 (2)	−0.008 (2)
C1	0.088 (5)	0.124 (6)	0.075 (4)	−0.022 (5)	0.017 (4)	0.006 (4)
C2	0.069 (4)	0.079 (4)	0.051 (3)	−0.011 (3)	0.007 (3)	−0.006 (3)
C3	0.081 (4)	0.074 (4)	0.047 (3)	−0.002 (3)	−0.004 (3)	0.006 (3)
C4	0.072 (3)	0.052 (3)	0.055 (3)	0.005 (3)	−0.010 (3)	−0.010 (2)
C5	0.064 (4)	0.131 (6)	0.088 (5)	0.002 (4)	0.009 (4)	−0.012 (4)
C6	0.074 (5)	0.156 (8)	0.097 (5)	−0.013 (5)	0.007 (4)	0.009 (5)
C7	0.111 (5)	0.057 (3)	0.055 (3)	−0.013 (4)	0.016 (4)	−0.009 (3)
C8	0.131 (6)	0.075 (4)	0.045 (3)	0.003 (4)	−0.001 (4)	0.004 (3)
C9	0.105 (5)	0.087 (4)	0.055 (4)	0.014 (4)	−0.017 (4)	−0.001 (3)
C10	0.133 (7)	0.167 (8)	0.076 (5)	0.032 (6)	−0.043 (5)	0.014 (5)
C11	0.097 (5)	0.103 (5)	0.082 (4)	0.014 (4)	−0.029 (4)	0.009 (4)
C12	0.156 (7)	0.120 (6)	0.073 (4)	−0.041 (6)	0.037 (5)	−0.003 (4)
O1W	0.033 (5)	0.055 (6)	0.122 (10)	0.000	−0.007 (6)	0.000

### Geometric parameters (Å, º)

**Table:**

Cu1—O2	1.897 (4)	C5—H5A	0.9700
Cu1—O1	1.901 (4)	C5—H5B	0.9700
Cu1—N2	1.922 (4)	C6—H6A	0.9700
Cu1—N1	1.926 (4)	C6—H6B	0.9700
O1—C2	1.291 (6)	C7—C8	1.398 (8)
O2—C9	1.287 (7)	C7—C12	1.509 (9)
N1—C4	1.301 (7)	C8—C9	1.352 (9)
N1—C5	1.454 (7)	C8—H8	0.9300
N2—C7	1.307 (7)	C9—C10	1.506 (9)
N2—C6	1.429 (8)	C10—H10A	0.9600
C1—C2	1.521 (8)	C10—H10B	0.9600
C1—H1A	0.9600	C10—H10C	0.9600
C1—H1B	0.9600	C11—H11A	0.9600
C1—H1C	0.9600	C11—H11B	0.9600
C2—C3	1.351 (7)	C11—H11C	0.9600
C3—C4	1.412 (7)	C12—H12A	0.9600
C3—H3	0.9300	C12—H12B	0.9600
C4—C11	1.514 (7)	C12—H12C	0.9600
C5—C6	1.431 (10)	O1W—H1WA	0.79 (2)
			
O2—Cu1—O1	86.59 (16)	H5A—C5—H5B	107.7
O2—Cu1—N2	93.75 (19)	N2—C6—C5	115.8 (6)
O1—Cu1—N2	177.56 (18)	N2—C6—H6A	108.3
O2—Cu1—N1	176.84 (19)	C5—C6—H6A	108.3
O1—Cu1—N1	93.48 (17)	N2—C6—H6B	108.3
N2—Cu1—N1	86.31 (19)	C5—C6—H6B	108.3
C2—O1—Cu1	125.8 (4)	H6A—C6—H6B	107.4
C9—O2—Cu1	126.2 (5)	N2—C7—C8	123.2 (6)
C4—N1—C5	121.5 (5)	N2—C7—C12	119.8 (7)
C4—N1—Cu1	126.2 (4)	C8—C7—C12	117.0 (6)
C5—N1—Cu1	112.3 (4)	C9—C8—C7	126.5 (6)
C7—N2—C6	122.7 (6)	C9—C8—H8	116.8
C7—N2—Cu1	125.4 (4)	C7—C8—H8	116.8
C6—N2—Cu1	112.0 (4)	O2—C9—C8	124.9 (6)
C2—C1—H1A	109.5	O2—C9—C10	114.5 (7)
C2—C1—H1B	109.5	C8—C9—C10	120.6 (6)
H1A—C1—H1B	109.5	C9—C10—H10A	109.5
C2—C1—H1C	109.5	C9—C10—H10B	109.5
H1A—C1—H1C	109.5	H10A—C10—H10B	109.5
H1B—C1—H1C	109.5	C9—C10—H10C	109.5
O1—C2—C3	125.9 (5)	H10A—C10—H10C	109.5
O1—C2—C1	113.6 (5)	H10B—C10—H10C	109.5
C3—C2—C1	120.5 (5)	C4—C11—H11A	109.5
C2—C3—C4	125.8 (5)	C4—C11—H11B	109.5
C2—C3—H3	117.1	H11A—C11—H11B	109.5
C4—C3—H3	117.1	C4—C11—H11C	109.5
N1—C4—C3	122.8 (5)	H11A—C11—H11C	109.5
N1—C4—C11	120.2 (5)	H11B—C11—H11C	109.5
C3—C4—C11	117.0 (5)	C7—C12—H12A	109.5
C6—C5—N1	113.2 (6)	C7—C12—H12B	109.5
C6—C5—H5A	108.9	H12A—C12—H12B	109.5
N1—C5—H5A	108.9	C7—C12—H12C	109.5
C6—C5—H5B	108.9	H12A—C12—H12C	109.5
N1—C5—H5B	108.9	H12B—C12—H12C	109.5

### Hydrogen-bond geometry (Å, º)

**Table:**

*D*—H···*A*	*D*—H	H···*A*	*D*···*A*	*D*—H···*A*
C12—H12*A*···O1*W*^i^	0.96	2.58	3.466 (9)	154
C6—H6*B*···O1*W*^i^	0.97	2.44	3.303 (11)	149
O1*W*—H1*WA*···O2	0.79 (2)	2.29 (14)	2.862 (8)	130 (13)

Symmetry code: (i) *x*−1/2, *y*−1/2, −*z*+1/2.
